# Missense Variant of Endoplasmic Reticulum Region of *WFS1* Gene Causes Autosomal Dominant Hearing Loss without Syndromic Phenotype

**DOI:** 10.1155/2021/6624744

**Published:** 2021-03-04

**Authors:** Jinying Li, Hongen Xu, Jianfeng Sun, Yongan Tian, Danhua Liu, Yaping Qin, Huanfei Liu, Ruijun Li, Lingling Neng, Xiaohua Deng, Binbin Xue, Changyun Yu, Wenxue Tang

**Affiliations:** ^1^Department of Otolaryngology Head and Neck Surgery, The First Affiliated Hospital of Zhengzhou University, Jianshedong Road No. 1, Zhengzhou 450052, China; ^2^Academy of Medical Science, Zhengzhou University, Daxuebei Road No. 40, Zhengzhou 450052, China; ^3^Precision Medicine Center, Academy of Medical Science, Zhengzhou University, Daxuebei Road No. 40, Zhengzhou 450052, China; ^4^The Second Affiliated Hospital of Zhengzhou University, Jingba Road No. 2, Zhengzhou 450014, China; ^5^Department of Bioinformatics, Technical University of Munich, Wissenschaftszentrum Weihenstephan, 85354 Freising, Germany; ^6^BGI College, Zhengzhou University, Daxuebei Road No. 40, Zhengzhou 450052, China; ^7^Henan Institute of Medical and Pharmaceutical Sciences, Zhengzhou University, Daxuebei Road No. 40, Zhengzhou 450052, China; ^8^The Third Affiliated Hospital of Xinxiang Medical University, Hualan Road, No. 83, Xinxiang 453000, China

## Abstract

**Objective:**

Genetic variants in the *WFS1* gene can cause Wolfram syndrome (WS) or autosomal dominant nonsyndromic low-frequency hearing loss (HL). This study is aimed at investigating the molecular basis of HL in an affected Chinese family and the genotype-phenotype correlation of *WFS1* variants.

**Methods:**

The clinical phenotype of the five-generation Chinese family was characterized using audiological examinations and pedigree analysis. Target exome sequencing of 129 known deafness genes and bioinformatics analysis were performed among six patients and four normal subjects to screen suspected pathogenic variants. We built a complete WFS1 protein model to assess the potential effects of the variant on protein structure.

**Results:**

A novel heterozygous pathogenic variant NM_006005.3 c.2020G>T (p.Gly674Trp) was identified in the *WFS1* gene, located in the C-terminal domain of the wolframin protein. We further showed that HL-related *WFS1* missense variants were mainly concentrated in the endoplasmic reticulum (ER) domain. In contrast, WS-related missense variants are randomly distributed throughout the protein.

**Conclusions:**

In this family, we identified a novel variant p.Gly674Trp of *WFS1* as the primary pathogenic variant causing the low-frequency sensorineural HL, enriching the mutational spectrum of the *WFS1* gene.

## 1. Introduction

Hearing loss (HL) is a very common sensory disorder and can be caused by genetic factors, viral infections, drugs, etc. [1]. More than 50% of prelingual HL cases are thought to be related to genetic factors [2]. The World Health Organization estimates that approximately 466 million people worldwide suffer from HL, including 34 million children (https://www.who.int/zh/news-room/fact-sheets/detail/deafness-and-hearing-loss). There are four genetic patterns of deafness: autosomal dominant (15–20%), autosomal recessive (80%), X-linked (1%), and mitochondrial DNA inheritance (1%). According to the presence of abnormalities in other organs, deafness is divided into syndromic (~30%) and nonsyndromic (~70%) HL. Deafness has extensive genetic heterogeneity [3], and 119 genes causing nonsyndromic HL have been identified (https://hereditaryhearingloss.org/). Different variants in the same gene, such as *CDH23* [4–6], *SLC26A4* [7, 8], and *WFS1* [9, 10], may lead to either syndromic or nonsyndromic HL.

The *WFS1* gene encodes a predicted 890-amino-acid transmembrane protein with a calculated molecular mass of approximately 100 kDa, which maps to chromosome 4q16. WFS1 is predicted to have nine central transmembrane domains, with an extracytoplasmic N terminus and an intracytoplasmic C terminus. In 1998, it was identified as the gene causing Wolfram syndrome, an autosomal recessive disorder, also called DIDMOAD (diabetes insipidus, diabetes mellitus, optic atrophy, and deafness) [11]. The deafness associated with DIDMOAD is characterized by high-frequency sensorineural HL. Some variants in this gene can also cause autosomal dominant deafness 6 (DFNA6), also known as DFNA14 or DFNA38, characterized by low-frequency sensorineural hearing impairment. WFS1 plays an important role in maintaining endoplasmic reticulum (ER) homeostasis, and pathogenic variants in WFS1 can lead to ER stress and cause early cell dysfunction and death [12, 13].

Next-generation sequencing technologies have been developed to detect the pathogenic variants underlying Mendelian disorders [14, 15]. We applied target exome sequencing of know deafness genes to determine the variants causing nonsyndromic postlingual deafness in a large Chinese family. A novel missense variant NM_006005.3 c.2020G>T (p.Gly674Trp) was identified in the *WFS1* gene that cosegregated with the HL phenotype. By querying the Human Gene Mutation Database (HGMD), ClinVar, and Deafness Variation Database (http://deafnessvariationdatabase.org/) and reviewing the literature, we found that *WFS1* missense variants correlated with HL and WS are different in the location relative to the protein domain.

## 2. Materials and Methods

### 2.1. Ethics Statement

The study was approved by the Committee of Medical Ethics of Zhengzhou University. Informed consent was obtained from all participants.

### 2.2. Clinical Evaluations

This study was conducted in a five-generation Chinese family from a village in Henan Province, China. This family has 65 members, including 11 with postlingual nonsyndromic HL. Among them, two patients had died and three patients refused to participate in this study; the remaining patients (II-2, III-2, III-8, III-10, III-12, and IV-3) and four normal subjects (III-1, III-3, III-11, and IV-4) underwent pure-tone audiometry (PTA), genetic test and clinical examinations, including internal auditory canal magnetic resonance imaging (MRI), computed tomography (CT) of the temporal bone, vision test, and fasting blood glucose level test.

### 2.3. Target Enrichment Sequencing

Genomic DNA was isolated from patient samples using the GenMagBio Genomic DNA Purification kit (GenMagBio, Changzhou, China) as per the manufacturer's standard procedures. Genomic DNA from affected and unaffected members was fragmented to an average size of 250 bp, and end repair, adapter ligation, and PCR enrichment were performed following the protocol of the VAHTS™ Universal DNA Library Prep Kit for Illumina V3 (Vazyme Biotech, Nanjing, China). Sequence capture was performed using the Human Deafness Panel oto-DA3 (Otogenetics, Atlanta, GA, USA), containing 129 known HL genes, following the manufacturer's protocol. The resulting libraries were sequenced on an Illumina HiSeq 4000 sequencer (Illumina, San Diego, CA, USA) with 150 bp paired-ends.

### 2.4. Bioinformatics Analysis and Variant Interpretation

Bioinformatics analysis and variant interpretation were carried out as described previously [16]. Sequencing adapters and low-quality reads were trimmed from the raw reads using Trimmomatic [17]. Clean reads were aligned to the human reference genome (ver. GRCh37) using the Burrow–Wheeler Aligner (ver. 0.7.17-r1188) [18], followed by duplicate reads marking using sambamba (ver. 0.6.6) [19]. Variant (single nucleotide variants and small indels) and genotype calling were performed using the Genome Analysis Toolkit ver. 4 (GATK4) HaplotypeCaller [20]. Variants were annotated by vcfanno [21] using the 1000 Genomes Project database [22], dbSNP [23], Exome Aggregation Consortium (ExAC) [24], Genome Aggregation Database (gnomAD) [25], ClinVar [24], InterVar [26], and dbNSFP [27]; the latter database compiled variant prediction scores from many prediction algorithms [28–30]. All analysis steps described above were performed in the framework of bcbio-nextgen (https://github.com/bcbio/bcbio-nextgen). We filtered out variants with minor allele frequencies > 0.05 in any general continental population, in which at least 2,000 alleles were observed in the gnomAD database, except those in the American College of Medical Genetics and Genomics (ACMG) benign stand-alone exception list or linked to diseases in the ClinVar database [31]. Variants were interpreted by an expert panel consisting of an otorhinolaryngologist, bioinformatician, and molecular geneticist following the guidelines of the ACMG and Association for Molecular Pathology for clinical sequence interpretation [32]. The variant nomenclature was based on the *WFS1* canonical transcript NM_006005.3.

### 2.5. Sanger Sequencing

To confirm candidate variants detected by next-generation sequencing, we performed PCR amplification and Sanger sequencing of the DNA from 10 individuals. Forward (5′-CCGGTGGTTCACGTCTCTGG-3′) and reverse (5′-GCCAGCAGCTTAAGGCGAC-3′) primers were designed using NCBI Primer-BLAST and synthesized by Shangya Biotechnology (Zhengzhou, China). PCR was conducted using the 2x Taq Master Mix kit (Novoprotein, Shanghai, China). The amplified products were subjected to 2.2% agarose gel electrophoresis, purified using a PCR purification kit (Lifefeng Biotechnology, Shanghai, China), and then sequenced using the SeqStudio Genetic Analyzer (Applied Biosystems/Life Technologies, Carlsbad, CA, USA).

### 2.6. Protein Structure Prediction of WFS1

The three-dimension (3D) model of the wolframin protein was built by a contact-assisted protein structure prediction method of CONFOLD2 [33], which leverages the information of interresidue contacts and secondary structures as well as energy functions. Based on the wolframin multiple sequence alignment (MSA) generated by HHblits [34], the interresidue contacts were then deduced from those residue pairs coupled evolutionarily by using CCMpred [35]. The secondary structures were predicted by SCRATCH1D [36]. We set for each threshold 5 best models and used the top-1 model selected by the final decision of CONFOLD2.

The reported missense variants at position 674 of the wolframin were used to perform in silico mutagenesis. The residue (Gly) was substituted to other four amino acids (Glu, Val, Arg, and Trp) as reported in the HGMD database, and an ensemble of the conformations (the number of conformations limits to 25) was generated for each mutant by low-mode MD (Molecular Dynamics). The force field used for calculation was OPLS-AA, and the implicit solvent was the reaction field (R-Field) model. All calculations were performed in Molecular Operating Environment (MOE) 2018 package [37]. Ab initio structure prediction of WFS1 was used to perform in silico mutagenesis.

## 3. Results

### 3.1. Clinical Features of the Family

**Figure 1:**
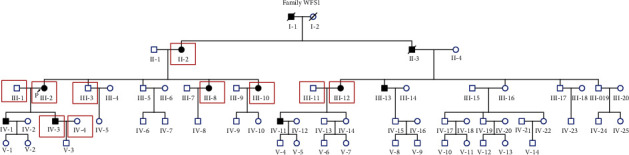
Pedigree of the family with autosomal dominant hearing impairment. Proband: arrow; affected individuals: gray symbols; males: squares; females: circles; participants clinically and genetically examined: red box.

**Figure 2:**
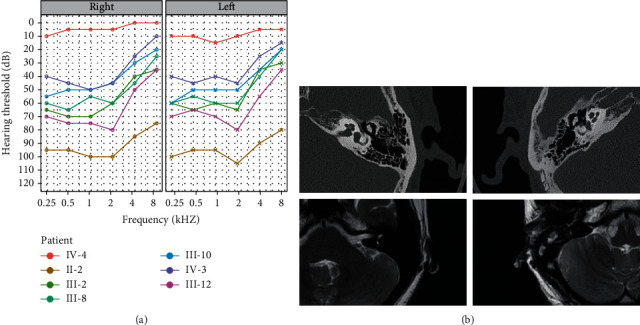
Audiological and imaging evaluation. (a) The PTA of the right and left ears of representative affected family members and a normal family member. (b) Internal auditory canal MRI and CT scans of the temporal bone in the proband (III-2). The first line contains two CT images of the proband's bilateral temporal bones. The second row contains two MR images of the proband's bilateral internal auditory canals.

The five-generation family has 11 affected individuals, ranging in age from 30 to 83 years (Figure 1). The family had a typical autosomal dominant inheritance pattern. In this family, the age of onset ranged from 20 to 30 years. The proband (III-2) developed moderate sensorineural HL involving low frequencies; the average auditory thresholds on PTA for the right and left ears were 67 and 63 dB HL, respectively (Figure 2(a)). In this family, subject II-2 is an 83-year-old lady, and her high-frequency hearing impairment might be related to presbycusis. The other affected members showed postlingual, bilateral nonsyndromic sensorineural HL involving low frequencies that was mild to profound. The patients had flat-sloping audiograms (Figure 2(a)). MRI and temporal bone CT in the proband showed a normal vestibular aqueduct and internal auditory canal (Figure 2(b)). No patient reported vestibular dysfunction, such as balance disorders, vertigo, or Meniere disease. All family members had normal blood glucose levels and visual function. There was no history of exposure to aminoglycosides or noise. Comprehensive examinations showed no evidence of DIDMOAD syndrome.  Table 1 shows the main clinical features of the affected individuals.

### 3.2. Target Enrichment Sequencing and Sanger Sequencing

**Figure 3:**
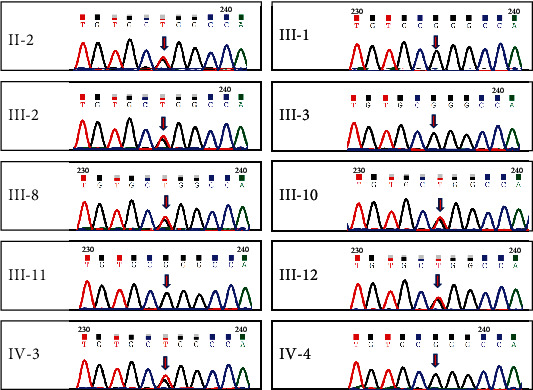
Sanger sequencing. Identification of c.2020G>T in the *WFS1* gene in six affected individuals (II-2, III-2, III-10, III-8, III-12, and IV-3); it was absent in the unaffected individuals.

Target exome sequencing of 129 known deafness genes was performed in six affected and four unaffected individuals (Figure 1) to identify potentially pathogenic variants. An average of one billion raw base pairs (1 Gbp) was generated for each sample, with more than 87% of the bases having a Phred quality score (*Q*) ≥ 30. The mapping rate to the GRCh37 human genome reference sequence exceeded 99.7%, and the average sequencing depth was 164.98x (range from 143.8x to 184.9x), covering 95.8% of the target regions at least 20x. The only variant in *WFS1*, NM_006005.3, c.2020G>T, cosegregated with HL in the family. Sanger sequencing of this variant in the family verified that the variant was present in the affected members and absent in the unaffected ones (Figure 3).

### 3.3. Variant Interpretation of the Variants as Pathogenic

The *WFS1* (NM_006005.3.) variant c.2020G>T (p.Gly674Trp) is present in exon 8, located in the C-terminal domain of the encoded wolframin protein. According to the standards and guidelines for interpreting genetic variants and the expert specification for genetic hearing loss proposed by the ACMG and the Association for Molecular Pathology [32, 38], this variant was classified as pathogenic (Table 2). This variant is absent from all population databases, including gnomAD and ExAC (Pathogenic Moderate 2, PM2). The variant cosegregated with the DFNA6/14/38 phenotype in the affected individuals (six affected, Pathogenic Supportive 1 Strong, PP1_Strong). The low-frequency HL in this family was highly specific for *WFS1* (Pathogenic Supportive 4, PP4). The REVEL score of this variant was ≥0.75 (Pathogenic Supportive 3, PP3). A known likely pathogenic variant (c.2021G>T, p.Gly674Val) is at the same amino acid residue with c.2020G>T (PM5). The evidence of p.Gly674Val is as follows: this variant was also absent from population databases (ACMG PM2), two probands with the variant (ACMG PS4_supporting) [10, 39], the variant segregated with the hearing impairment in a Dutch family (ACMG PP1) [10], REVEL score ≥ 0.75 (ACMG PP3), and patient's phenotype or family history highly specific for a disease with a single genetic etiology (ACMG PP4).

### 3.4. Missense Variants Leading to HL and WS in Wolframin Protein

**Figure 4:**
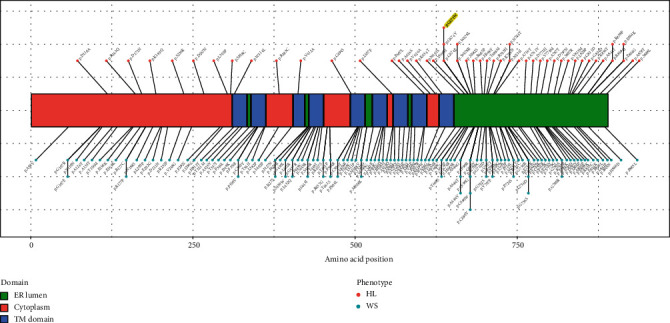
The distribution of missense variants in wolframin protein leading to HL and Wolfram syndrome. The pathogenic missense variants associated with HL or Wolfram syndrome were collected from the Human Gene Mutation Database, ClinVar, and Deafness Variation Database. Missense variants in the *WFS1* gene leading to HL are located mainly in the ER lumen domain. The highlighted p.G674W is a novel *WFS1* variant reported in this study. Endoplasmic reticulum lumen: ER lumen; transmembrane domain: TM domain; hearing loss: HL; Wolfram syndrome: WS.

Missense variants in wolframin protein can cause both WS and nonsyndromic HL. We classified these variants of *WFS1* according to different phenotypes and drew a distribution map of these variants in wolframin protein. We found that the missense variants that caused WS were distributed in all protein regions, while the missense variants that caused HL were clustered in the ER lumen domain (Figure 4).

**Figure 5:**
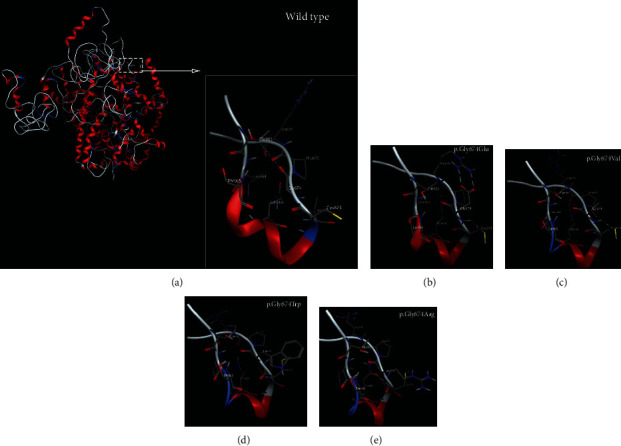
3D protein modeling of WFS1 variants at amino acid residue 674 leading to hearing loss or Wolfram syndrome. (a) Wild type has a hydrogen bond between Gly674 and Thr663; (b) p.Gly674Glu. The variant alters the interaction between Glu674 and Thr663 and increases two hydrogen bonds between Glu674 and Arg676, a hydrogen bond between Glu674 and Cys673, and a hydrogen bond between Gln668 and Pro675; (c) p.Gly674Val. The variant increases a hydrogen bond between Val674 and Cys673, and a hydrogen bond between Gln668 and Pro675; (d) p.Gly674Trp. The variant increases a hydrogen bond and two arene interactions between Trp674 and Cys673 and a hydrogen bond between Gln668 and Pro675; (e) p.Gly674Arg. The variant increases a hydrogen bond between Gln668 and Leu672 and two hydrogen bonds between Gly674 and Cys673.

To reveal the potential mechanism of different missense variants at the same protein position lead to varying diseases, we assessed the potential effects of variants on protein structure by taking four missense variants (nonsyndromic HL: p.Gly674Trp, p.Gly674Glu, and p.Gly674Val; WS: p.Gly674Arg) as an example. We built a complete WFS1 protein model from scratch as there are no three-dimensional molecular structures for any parts of WFS1 available. The three-dimensional structure revealed that all of the four missense variants cause loss of the hydrogen bond interaction between p.Gly674 and p.Thr663, which was observed in wild type. Notably, compared with WS-associated p.Gly674Arg, all the three HL-related variants increased an extra hydrogen bond between p.668Gln and p.675Pro (Figure 5). Changes in hydrogen bonds may affect the stability of the WFS1 protein, suggesting a potential molecular mechanism resulting in varying diseases.

## 4. Discussions

We examined a five-generation Han Chinese family with postlingual nonsyndromic autosomal dominant HL using target sequencing of 129 genes associated with HL. Extensive clinical evaluation of the family before molecular genetic analysis confirmed nonsyndromic HL with no associated features that segregated with the HL. A novel *WFS1* c.2020G>T (p.Gly674Trp) missense variants was the only variant cosegregating with HL identified in this family. We further showed that compared with missense variants associated with WS, HL-related ones are mainly located in the ER domain.

*WFS1* encodes a transmembrane protein (wolframin) that is located primarily in the ER; it was highly expressed in the brain, pancreas, heart, and insulinoma beta-cell lines [40] and differentially expressed in inner ear cells [41]. Wolframin plays a role in protein folding [42] and negatively regulates ER stress [13], maintaining endolymphatic ion homeostasis. Once a variant occurs in *WFS1*, the negative regulation of a feedback loop in the ER stress signaling network disappears, resulting in the accumulation of misfolded and unfolded proteins, leading to cell death [43, 44]. In the inner ear, mutated WFS1 may alter intracellular Ca^2+^ homeostasis [42], the recycling of K^+^ back to the endolymph [45], and other endolymphatic ions. p.Gly674Trp is located in the ER lumenal domain. Glycine is most often replaced by charged amino acids, but the location of the variant rather than the properties of the substituting amino acid has a greater impact on disease severity [46]. We speculate that the variants strongly induce ER stress in inner ear cells and disrupt endolymphatic ion composition and homeostasis, which is the leading cause of deafness.

Different variants in *WFS1* have different effects on wolframin. Hofmann et al. first show that missense variants do not affect the stability of *WFS1* mRNA, but the wolframin_R629W_ protein level was reduced substantially compared with wild-type wolframin [47]. Different amino acid substitutions at the same site can cause different phenotypes. For example, different substitutions at amino acid 678 of *WFS1* resulted in three distinct phenotypes: sensorineural HL [48, 49], Wolfram syndrome [50], and optic atrophy and diabetes [51]. The missense variants c.2032T>C (p.Trp678Arg) and c.2033G>T (p.Trp678Leu) lead only to sensorineural HL, whereas the nonsense variants c.2033G>A (p.Trp678Term) and c.2034G>A (p.Trp678Term) lead to more severe symptoms of Wolfram syndrome or optic atrophy and diabetes. The novel missense variant c.2020G>T (p.Gly674Trp) identified in this study caused only nonsyndromic HL in the family. Interestingly, a change from glycine to arginine at the same amino acid position (c.2020G>A, p.Gly674Arg) led to a severe Wolfram syndrome phenotype [52]. These findings can contribute to a better understanding of different variants that lead to phenotypes of different severities.

In conclusion, we identified a novel variant c.2020G>T (p.Gly674Trp) in *WFS1* responsible for autosomal dominant low-frequency sensorineural HL in a Chinese family, extending the variant spectrum of *WFS1*. Based on the variant location in the ER domain, we speculate that the variant might cause hearing impairment due to ER stress. Due to the instability of inner ear cells *in vitro*, the mechanism of low-frequency sensorineural HL caused by *WFS1* variants is unclear. Further studies should explore which domains of the wolframin protein function in hearing and determine the pathogenic effects of genetic variants on the protein.

## Figures and Tables

**Table 1:** Summary of the clinical data from the affected individuals in the family.

Subject no.	Gender	Age	Age of onset	Usage time for HA	Audiogram shape	Tinnitus	Vertigo
II-2	Female	83	22	20	Flat-sloping	Yes	No
III-2	Female	60	20	8	Flat-sloping	Yes	No
III-8	Female	55	30	4	Flat-sloping	No	No
III-10	Female	57	25	7.5	Flat-sloping	No	No
III-12	Female	63	20	11	Flat-sloping	Yes	No
IV-3	Male	30	19	8	Flat-sloping	Yes	No

HA: hearing aid.

**Table 2:** The candidate variant identified in the family.

Gene symbol	Nucleotide change	Amino acid change	SIFT	PolyPhen-2	MutationTaster	Pathogenicity	ACMG evidence
*WFS1*	c.2020G>T	p.Gly674Trp	Damaging	Damaging	Damaging	Pathogenic	PM2; PM5; PS4_S; PP1_S; PP3; PP4

PP1_S: PP1_Strong; PS4_S: PS4_Supporting.

## Data Availability

All data can be viewed in NODE (http://www.biosino.org/node) by pasting the accession (OEP001069) into the text search box or through the URL: https://www.biosino.org/node/project/detail/OEP001069.

## Abbreviations

HL: Hearing loss
ER: Endoplasmic reticulum
PTA: Pure-tone audiometry
MRI: Magnetic resonance imaging
CT: Computed tomography
DIDMOAD: Diabetes insipidus, diabetes mellitus, optic atrophy, and deafness
DFNA6/14/38: Autosomal dominant deafness 6/14/38
ACMG: American College of Medical Genetics and Genomics
HGMD: Human Gene Mutation Database
MOE: Molecular Operating Environment
MD: Molecular Dynamics.
